# Diversity Indices of Plant Communities and Their Rhizosphere Microbiomes: An Attempt to Find the Connection

**DOI:** 10.3390/microorganisms9112339

**Published:** 2021-11-12

**Authors:** Aleksei O. Zverev, Arina A. Kichko, Aleksandr G. Pinaev, Nikolay A. Provorov, Evgeny E. Andronov

**Affiliations:** 1 All-Russian Research Institute for Agricultural Microbiology (ARRIAM), Podbelsky Chaussee 3, 196608 St. Petersburg, Russia; 2014arki@gmail.com (A.A.K.); ag.pinaev@gmail.com (A.G.P.); provorovnik@yandex.ru (N.A.P.); eeandr@gmail.com (E.E.A.); 2 Dokuchaev Soil Science Institute (SSI), Pyzhevskiy Pereulok 7, 119017 Moscow, Russia

**Keywords:** diversity correlation, alpha-diversity of plants, ITS1 plants sequencing, crop rhizosphere, 16S and ITS1 sequencing

## Abstract

The rhizosphere community represents an “ecological interface” between plant and soil, providing the plant with a number of advantages. Despite close connection and mutual influence in this system, the knowledge about the connection of plant and rhizosphere diversity is still controversial. One of the most valuable factors of this uncertainty is a rough estimation of plant diversity. NGS sequencing can make the estimations of the plant community more precise than classical geobotanical methods. We investigate fallow and crop sites, which are similar in terms of environmental conditions and soil legacy, yet at the same time are significantly different in terms of plant diversity. We explored amplicons of both the plant root mass (ITS1 DNA) and the microbial communities (16S rDNA); determined alpha- and beta-diversity indices and their correlation, and performed differential abundance analysis. In the analysis, there is no correlation between the alpha-diversity indices of plants and the rhizosphere microbial communities. The beta-diversity between rhizosphere microbial communities and plant communities is highly correlated (R = 0.866, *p* = 0.01). ITS1 sequencing is effective for the description of plant root communities. There is a connection between rhizosphere communities and the composition of plants, but on the alpha-diversity level we found no correlation. In the future, the connection of alpha-diversities should be explored using ITS1 sequencing, even in more similar plant communities—for example, in different synusia.

## 1. Introduction

As the formation of a specific microbial communities near the plant root, the phenomenon of the rhizosphere effect has been the subject of many works of both classical and modern biology. The rhizosphere community represents an “ecological interface” between plant and soil, providing the plant with a number of advantages, such as growth stimulation, protection from pathogens, and nutrition, among others [1,2]. The source of the rhizosphere microbiome is both the microbial community of plant seeds [3] and the community of soil microorganisms [4]. The composition and abundance of plant root exudates determines the formation of the bacterial community [5,6]. The source of the microbiome, and the development of it, thus forms the final community.

The diversity of sources and the different ways of development lead to the specificity of the rhizosphere microbiome. The composition of rhizosphere communities can be affected by several factors. In addition to the type and agrochemical properties of the soil, the genotype of the plant (species [7,8] and cultivar [9]) is a significant factor for microbiome development. The genotype factor can be explained by a specific exudation spectrum from various plants. The spectrum of secreted substances depends not only on the species or cultivar but also on the developmental phase, physiological state, etc. [10,11]. In turn, the microbial communities themselves affect the metabolic status of the plant [12], which allows us to talk about the “plant-rhizosphere community” system as a self-adjusting one (a kind of “gut-brain axis” in plants). This system is additionally complicated by the high diversity of plant species, which is natural in indigenous plant populations.

Diversity estimation is one of the ways of describing both bacterial and plant communities. As self-adjusting systems, rhizosphere microbiomes and plant communities are connected, and therefore, the following question arises: is the diversity of bacterial communities related to the diversity of plant communities? Based on the literature, there are two assumptions: (1) an increase in the species richness of plants leads to an increase in the number of available micro-niches, which leads to an increase in microbial diversity; and (2) an increase in the species richness of plants is accompanied by the predominant development of bacteria from highly productive specific taxa, which leads to a general decrease in the diversity of microorganisms [13]. Experimental studies of this relationship show controversial results: some studies indicate the absence or negative correlation between plant and bacterial richness [14,15], whereas others show the presence of a positive relationship [16]. Beta diversity indices show an unambiguous positive correlation between the distances of plant and bacterial communities [17].

One of the reasons for the uncertainty in this area is, most likely, the problem of the correct estimation of plant diversity. The development of NGS methods for the estimation of microbial diversity, from DNA extraction methods to statistical analysis of libraries (16S, ITS), has led to the rise of this area observed today [18]. However, in most papers, plant diversity is determined using geobotanical methods [13,15]. This approach is not accurate enough in both determining the species in the communities and their abundances [19]. In addition, in the context of the aim of this study, there is a question about the correspondence between the “aboveground” and “belowground” plant diversities, which can be quite different [20].

Experiment design is another significant point of connection research. The rhizosphere community is highly variative and is influenced by different soils, tillage or nutrition [11]. Therefore common soil legacy is important for the investigation of possible correlation. A reasonable solution is to analyze different plant communities from the same geographic location. In this research we use rye crop fields (as low diverse plant community) and fallow sites (as high diverse plant community) located nearby.

Contrary to previously published research, we are going to focus on two main points: (1) to analyze the diversity of the different plant populations with the same soil legacy (same location, soil type, water regime, etc.); and (2) to analyze plant communities via direct DNA isolation from the plant root mass followed by NGS sequencing of the ITS1 region.

This approach allows us to (1) characterize plant communities via their underground parts (which interact with rhizosphere microorganisms); (2) use the same sample for both plant and microbiome analysis (therefore, each rhizosphere library matches with the plant library); and (3) use standard approaches for processing and calculating diversity indices and downstream analysis.

We hypothesize that this scrutiny can allow us to find positive or negative correlations between the alpha-diversity of plant communities and their rhizosphere microbial communities.

## 2. Materials and Methods

### 2.1. Sampling

Samples were collected on 21 July 2017 on the fields of the Pskov Research Institute of Agricultural Sciences and Rodina State Farm in the Pskov region, Russia. We selected one site within a rye crop field (referred to as Monoculture Rye or MonoR—57.845638 N 28.201125 E) and fallow sites outside the field border from two locations, dominated by cereals (Polyculture Cereal or PolyC—57.845519 N 28.201263 E) and *Galium* and *Dactylis* species (Polyculture Galium, or PolyG—57.845589 N, 28.201708 E). In each site, three samples were taken up to 2 m from center point. The geobotanical description of the sampling sites is provided in the Supplementary Material.

Bricks of topsoil (about 15 × 15 × 10 cm) were cut (by shovel and knife) from a soil surface and placed in individual zip-lock packages. In less than 48 h all packages were transferred into a laboratory. In the laboratory, the bulk soil was gently removed manually and by shaking, 30 g of the root mass was intensively shaken with 50 mL of 0.005 M Na-phosphate buffer in a Pulsifier II (Microgen, Camberley, UK) in provided bags (PUL512 Bags) for 1 min. The liquid fraction was centrifuged, and the pellet was used to isolate the total rhizosphere DNA. The root mass was used to isolate plant DNA.

### 2.2. DNA Extraction and Sequencing

Procaryotic DNA from the pellet was isolated using the MN NucleoSpin Soil Kit (Macherey-Nagel, Dueren, Germany) and a Precellus 24 homogenizer (Bertin, Montigny-le-Bretonneux, France) according manufacturer protocol. Quality control was carried out by PCR and agarose gel electrophoresis. Sequencing of the V4 variable region of the 16S rRNA gene was performed on an Illumina MiSEQ sequencer (Illumina Inc., San Diego, CA, USA), using the primers F515 (GTGCCAGCMGCCGCGGTAA) and R806 (GGACTACVSGGGTATCTAAT) [21].

Plant DNA from roots was isolated using mechanical destruction in liquid nitrogen, followed by CTAB extraction [22]; the quality of the DNA was also checked via agarose gel electrophoresis. Sequencing of the ITS1 variable region was performed on an Illumina MiSEQ sequencer (Illumina Inc., San Diego, CA, USA), using the primers ITS-p5 (YGACTCTCGGCAACGGATA) and ITS-u2 (GCGTTCAAAGAYTCGATGRTTC) [23].

### 2.3. Bioinformatic Processing

The general processing of sequences was carried out in R 3.6.4, using the dada2 (v. 1.14.1) [24] and phyloseq (v. 1.30.0) [25] packages, according authors’ recommendations.

16s amplicone sequences were processed according to the dada2 pipeline. Sequences were trimmed by length (minimum 220 bp for forward and 180 bp for reverse reads) and quality (absence of N, maximum error rates maxEE were 2 for both forward and reverse reads). An ASV was determined according dada2 algorithm, and chimera ASVs were removed by “consensus” method. Taxonomic annotation was performed by the naive Bayesian classifier (provided in dada2 package, default settings) with the SILVA 138 database [26] as the training set.

ITS1 sequences were processed according to dada2 pipelines for ITS region. Primer sequences were identified and removed by cutadapt [27]. Sequences were trimmed by length (minimum 50 bp for both reads) and quality (absence of N, maximum error rates maxEE were 2 for both forward and reverse reads). ASVs were determined according to the dada2 algorithm (version for ITS); chimera ASVs were removed by “consensus” method. Taxonomic annotation was performed by the naive Bayesian classifier (provided in dada2 package, default settings) with the PLANiTS [28] database as the training set.

The main alpha- and beta-metrics were calculated using the phyloseq and picante [29] packages. For the mean p-distance, we used the home-brew script (calculated as sum of weighted pairwise p-distance from multiple alignment). Correlations between diversity indices were calculated using the Spearman correlation. Significant differences in abundances of taxa between sites were determined using theDeSEQ2 package [30].

All reads were submitted to SRA (PRJNA649486) and are available under the link https://www.ncbi.nlm.nih.gov/sra/PRJNA649486 (Submitted 29 July 2020).

## 3. Results

The ITS1 sequencing of plant DNA yielded 230 ASVs. The taxonomic position at genus level was defined for 217 of them; the number of reads per sample after rarefaction was 14,210. Regarding 16s rDNA, we found 5284 ASVs, with 15,487 reads per sample. Also worth mentioning is that despite the F515/R806 primers specific for both Bacteria and Archaea, the Archaea amount was less than 0.1% and wasn’t significant in downstream analysis.

### 3.1. Taxonomic Composition

**Figure 1:**
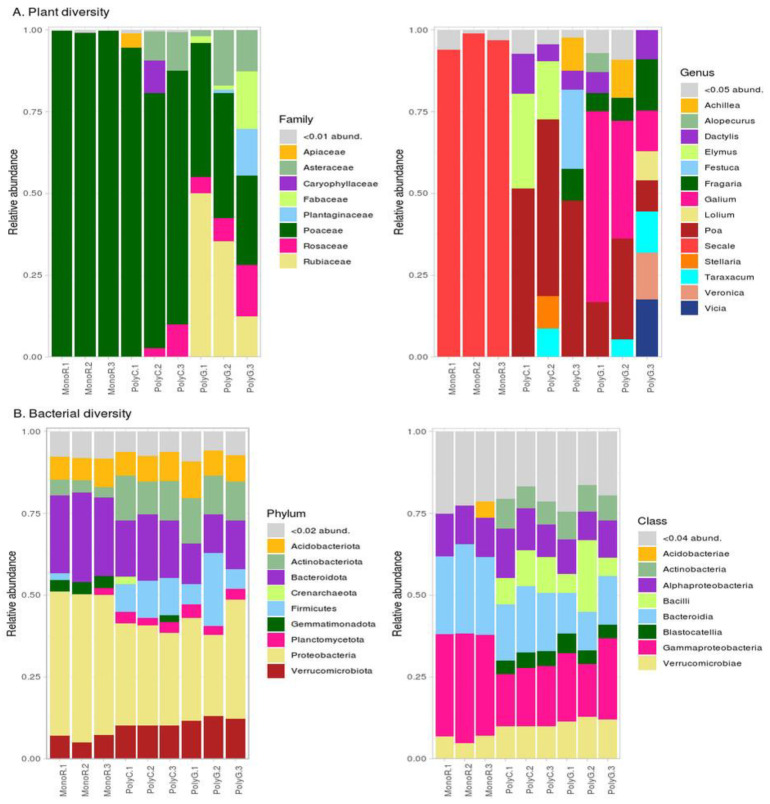
Relative abundances of plant and bacterial communities in the experimental sites. (**A**): Plant diversity; (**B**): Bacterial diversity.

Figure 1 shows the taxonomic composition of the communities. The geobotanic description (provided in the Supplementary Section) corresponded with the composition structure according to ITS1 sequencing (Figure 1A). Almost all reads from MonoR libraries were attributed as Secale; PolyC (samples from the cereal synusia) had about half sequences from Poa genus, followed by Elymus and Dactilus. PolyG (samples from Galium and Dactylis synusia) was more diverse; most reads were attributed as Galium, Poa. Despite the geobotanical description of this synusia as mixed Galium and Dactylis, there was no evidence of a great amount of Dactylis in the PolyG libraries, which can be explained by difficulties in describing the graminae vegetation outside the flowering phase.

**Figure 2:**
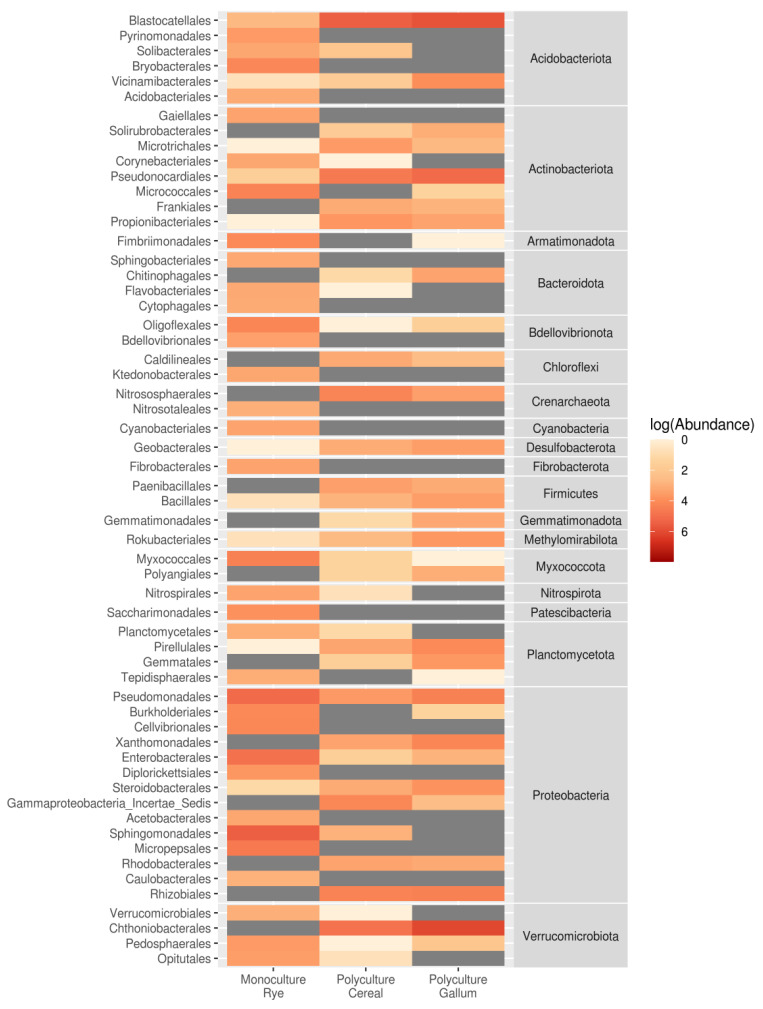
Mean abundances of bacterial orders with different inter-source abundances (according to DeSEQ2).

The bacterial communities (Figure 1B) were typical for rhizosphere microbiomes [11]. The communities from the two fallow sites (PolyC, PolyG) were similar, whereas the communities from the rye site (MonoR) contained more Gammaproteobacteria (Proteobacteria) and Bacteroidia (Bacteroideta). Differential abundance analysis (with information about abundance in repeats) allowed us to find 584 microbial taxa, which significantly differed between crop and fallow sites. In comparison with PolyC and PolyG, none of the ASVs was marked as significantly different. The results of the pairwise comparison of all three sites are shown in Figure 2 at the order level.

In this comparison, the most abundant ASVs in rye crop rhizosphere microbiomes were from Proteobacteria (orders Pseudomonadales, Sphingomonadales, Enterobacteriales), followed by Armatimonadota (Fimbriimonadales), Acidobacteriales (Bryobacterales), and Actinobacteriota (Micrococcales). In fallow root microbiomes, Verrucomicrobia (Chthoniobacteriales), Acidobacteria (Blastocatellales), and Actinobacteria (Pseudonocardiales) were more abundant.

### 3.2. Alpha-Diversity Indices

**Figure 3:**
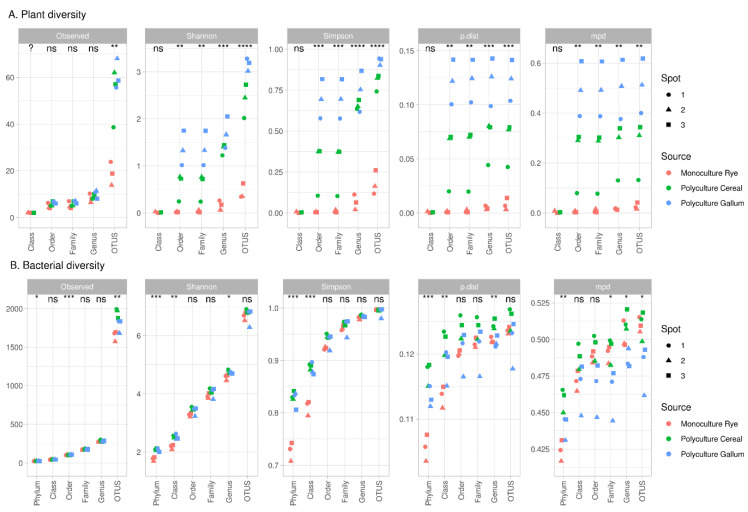
Alpha-diversity indices for plant and bacterial communities (significance for ANOVA in groups; ?—not available, ns—non-significant, *—*p* < 0.05, **—*p* < 0.01, ***—*p* < 0.005, ****—*p* < 0.001). (**A**): Plant diversity; (**B**): Bacterial diversity.

For all samples, the most common alpha-diversity indices, observed ASVs, Shannon, Simpson, mpd (mean pairwise distance), and p-dist (mean p-distance, restored from alignment, see Materials and Methods), were calculated at different taxonomic levels (Figure 3).

The plant communities were different (Figure 3A). Unweighted indices, as the observed ASV index, was not useful because of the presence of weedy plants in the rye crop. Weighted indices, such as mpd or p-dist, were more suitable; the differences between sample sites were significant for both indices up to the order level.

The bacterial communities were less diverse. The observed ASVs as well as the p-dist and mpd indices showed that communities from the PolyC site were more diverse, mostly at low taxonomical levels (ASVs, genus). Interestingly, samples from rye roots (MonoR) were significantly less diverse at phylum level, whereas at other taxonomical ranks, there were no such differences.

For each index on the ASV taxonomic level, the correlation between plant diversity indices and microbial diversity indices was calculated. We observed no significant correlations between plant and rhizosphere microbial diversity.

### 3.3. Beta-Diversity Indices

**Figure 4:**
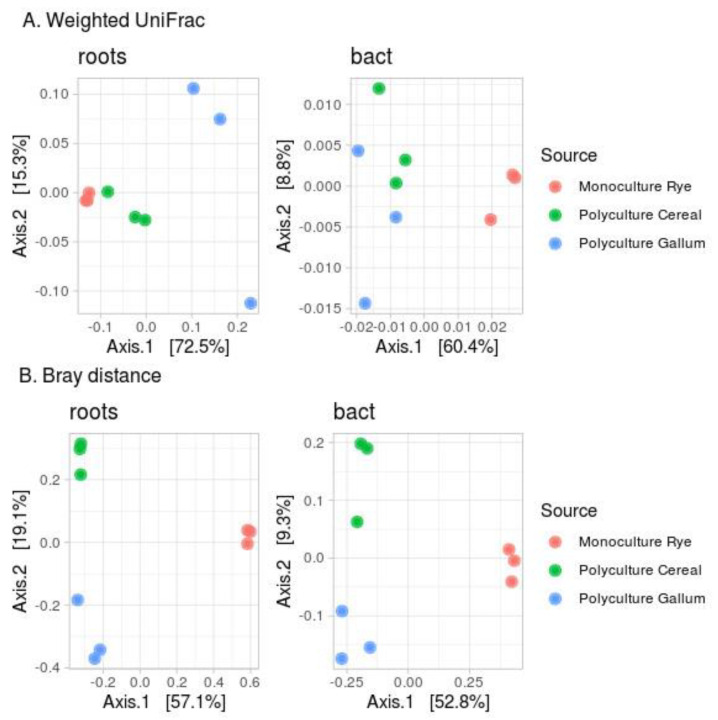
Beta −diversity indices for plant and bacterial communities. (**A**): Weighted UniFrac; (**B**): Bray distance.

Figure 4 shows the beta-diversity indices (Bray distance and weighted UniFrac). According to the weighted UniFrac index (Dunn index for roots 0.316, for microorganisms 0.821), the samples were mixed (Figure 4A). Samples from rhizosphere communities from PolyC and PolyG were mixed in one cluster. While the MonoR and PolyC samples were close, the PolyG samples were different. This might be connected to the phylogenetic composition of the population; Secale and Poa, the two main genera from these sites, both belong to the Poaceae family, whereas Galium belongs to the Rubiaceae family (Figure 1A).

According to the Bray distance (Dunn index for roots 0.832, for microorganisms 0.909), all communities, from plants and from rhizospheres, formed their own clusters (Figure 4B).

**Figure 5:**
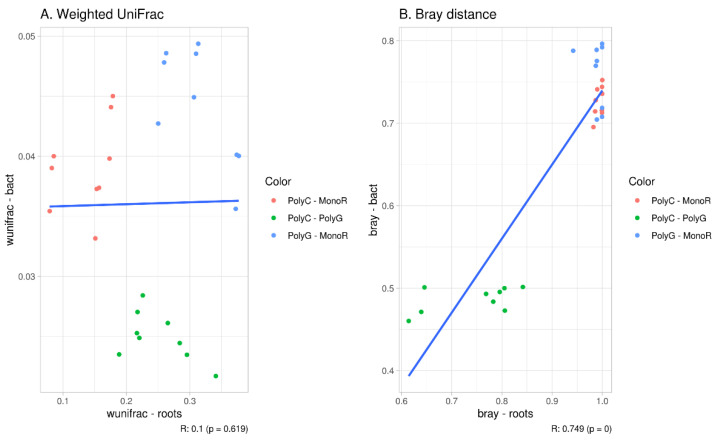
Correlation between beta-diversity indices. (**A**): Weighted UniFrac; (**B**): Bray distance.

The correlation between the distance matrix in plant and bacterial communities was high for the Bray distance (R = 0.866, *p* = 0.01) and not significant for the weighted UniFrac index. Figure 5 shows the results of the beta-distance correlation, excluding the intra-cluster distance (distance between repeats of the same sample).

## 4. Discussion

The aim of this work was to determine the potential connection between the diversity of plant communities and the diversity of rhizosphere microbiomes. For this purpose, we used three sites with different plant communities in the same location: a rye crop monoculture (MonoR) and two polyculture fallow sites, dominated by cereals (PolyC) and Galium and Dactylis (PolyG). Soil type, water regime, and main soil properties were similar for all three sites, which minimized the influence of these factors on microbial communities.

The novel ITS1 sequencing is highly effective for the estimation of plant community composition. Almost all (217 of 230) determined ASVs were annotated, and the composition and abundance of plant genera fit the geobotanical description.

For samples obtained from same soil and location, the most important factor shaping the microbial community of the rhizosphere is the plant community structure. We hypothesize that the variation in the spectrum of plant exudates could be one of the possible mechanisms responsible for the diversity of the rhizosphere microbial community. This spectrum is specific for plant species and genotypes and, therefore, in the case of the whole plant community, we can talk about an “exudome” of the community. The structure of this exudome depends on the abundance of different plants in the community. In the comparison of two polycultures, the number of plant species is the same (as shown in Figure 3A), with variations in their abundance; it therefore seems logical that the exudomes of these communities also will differ not insignificantly. In contrast, when comparing monoculture plant communities with polyculture ones, there are differences in the plant composition. In this case, exudomes of plant communities will differ.

In this study, using modern approaches, we found no correlation between different alpha-diversity indices. According to all indices, rhizosphere communities in the monoculture site (MonoR) were as diverse as those in the polycultures (PolyR, PolyC), whereas the plant community diversity in the monocultures was obviously lower. Meanwhile, in the polyculture samples (in mpd and p-dist indices) more diverse plant communities corresponds with a less diverse rhizosphere microbial community. This might be evidence of a negative connection, as suggested by Goberna and co-authors [13], but this connection, presumably, can be found only in plant communities with similar exudomes (plant communities with a different abundance of plants, not by plant composition itself). Significant differences in exudomes can lead to different microbial response and a loss of similarity when different pathways and intermediates completely reshape the community.

This idea also fits well with the beta diversity plots and correlations. Indeed, the distances between samples from polycultures were small for both plant and bacterial communities (about 17% of the explained variance in PcoA plots for plants and 8% for microorganisms). The distances between samples from mono- and polycultures were large (about 60% of the explained variance in PcoA plots). This allowed us to estimate a correlation for Bray distances (due to the taxonomic similarity between rye and cereal, the correlations between weighted Unifac distances were not significant). This correlation might be a true correlation, as observed in previous studies [17], or a statistical artifact (caused by the large distance between monoculture and polyculture clusters).

Despite the expected main difference in plant diversity between poly- and monocultures, with the moderate differences between polycultures, both alpha- and beta-diversity of the plants showed significant variations between synusiae. In the community of different cereals, PolyG samples in most cases were significantly more diverse by all alpha-diversity indices, and showed inter-sample variability (sample PolyG.3). This effect is also clear at higher taxonomic levels (family or order).

The differential abundance analysis of microbial communities did not show any difference between rhizosphere communities of polycultures. Similarly, ASVs and Shannon indices of these samples were also not significantly different. The phylogenetic-related indices (mpd and p-dist) were different, although this can be both a bias of a method or possible difference in the phylogenetic position of the bacteria. Further elaboration of this theory is recommended.

In contrast, in comparison of microbial communities of the polyculture and monoculture sites, there was a significant difference in the observed ASVs, whereas phylogenetic-related indices (mpd, p-dist) showed no clear pattern. Differential abundance analysis allows us to identify taxa with significant differences in relative diversity. Some of them have previously been reported as rhizosphere taxa. Sphingomonadales and Enterobacteriales are typical for plant rhizosphere communities [31], and Fimbriimonadales has previously been described as a bacterial taxon from the *Anthurium andraeanum* rhizosphere community [32]. In the same case, Blastocatellales has previously been described as an oligotrophic, slightly acidophilic to neutrophilic mesophile from arid soils [33]. However, these data are controversial, as they can describe a novel location of this microorganism or a biased database. More precise data can be obtained by functional or full-genomic analyses of rhizosphere communities.

A closer interaction could be revealed with the use of genes directly involved in plant-rhizosphere communication processes. These could be plant genes involved in plant root exudation and responses to bacterial signals (for example, strigolactone-processing genes), and bacterial genes involved in the decomposition of those exudates by microorganisms. Also, it is important to characterize and compare the structure and composition of exudation profiles of different plant communities.

## 5. Conclusions

Sequencing of the ITS1 region is highly effective for the taxonomical annotation of plant communities. Almost all ASVs were attributed, and abundances of taxa fairly corresponded to the geobotanical descriptions.

Despite using NGS tecnology, the connection between the composition of plants and their rhizosphere communities is still discordant. We found a strong correlation in the beta-diversity of plant-bacteria communities, but not in alpha-diversity. The bacterial taxa, responsible for the difference in rhizosphere communities, are rhizosphere-connected taxa.

According our results, the matter of alpha-diversity connection should be investigated more thoroughly. We suggest the exploring of this connection using very close plant communities (like synusia).

Plants are one of the most important environmental factors for soil microorganisms, therefore, our research on diversity assessing can be useful for the researchers of ecological connections. In future studies, the diversity connection should be analyzed by NGS plant sequencing. Another direction is the searching for and analysis of functionally related genetic regions of plants and soil microorganisms.

## Data Availability

Seqences are avalible at SRA by access number PRJNA649486 or by the link: https://www.ncbi.nlm.nih.gov/sra/PRJNA649486 (Submitted 29 July 2020).

## Supplementary Materials

The following are available online at https://www.mdpi.com/article/10.3390/microorganisms9112339/s1, Figure S1: General view, current location and root samples. MonoR-rye crop field; PolyC-421 forb and cereal meadow dominated by cereals; PolyG-forb and cereal meadow dominated by *Galium* and *Dactylis* species. Samples are: 1—MonoR.1; 2—MonoR.2; 3—MonoR.3; 4—423 PolyG.1; 5—PolyG.2; 6—PolyG.3; 7—PolyC.1; 8—PolyC.2; 9—PolyC.3. Table S1: Geobotanical description of the sampling sites.
