# Supplementary material for: Diversity Indices of Plant Communities and Their Rhizosphere Microbiomes: An Attempt to Find the Connection
Source: Microorganisms. 2021 Nov 12;9(11):2339. doi: 10.3390/microorganisms9112339 (PMC8619031; doi:10.3390/microorganisms9112339)
Supplement: Supplementary file 1 [file microorganisms-09-02339-s001.zip › S1_Table.pdf]

Table S1: Geobotanical description of the sampling sites.

	**Rye field description**	**Fallow site description**
	Rye crop field. The crops are uniform, of good quality, without visible signs of diseases and pests. The height of rye plants is 1.5–1.65 m, and the total vegetation cover is 85–90%, with 10–15% bare soil between rows.	Forb and cereal meadow. Agricultural land previously used as hayfield. Over the past several years, haymaking and other activities have not been carried out. The grass is high, dense, 100% closed. The height of the generative parts of plants reaches 1.0 m. There are two synusiae in the meadow. Main synusia formed by *Galium* and *Dactylis* (referred to as PolyG); smaller areas are occupied by cereal synusia, with the co-dominance of several species of cereals such as *Dactylis*, *Bromopsis*, and *Alopecurus* (referred to as PolyC).
**Genus**	**Plant abundance on rye field**	**Plant abundance on fallow site**
Achillea		<1
Alchemilla		5
Alopecurus		3
Bromopsis		7
Centaurea		1
Cerastium	5	
Dactylis		15–50
Fragaria		7–12
Galium	<1	5–20
Myosotis	<1	
Plantago	<1	
Poa	7	
Secale	80–85	
Stellaria	5	5
Taraxacum		7
Trifolium		3

Vicia		7
Viola	1–2	
