# Supplementary figures and images for: Diversity Indices of Plant Communities and Their Rhizosphere Microbiomes: An Attempt to Find the Connection
Source: Microorganisms. 2021 Nov 12;9(11):2339. doi: 10.3390/microorganisms9112339 (PMC8619031; doi:10.3390/microorganisms9112339)

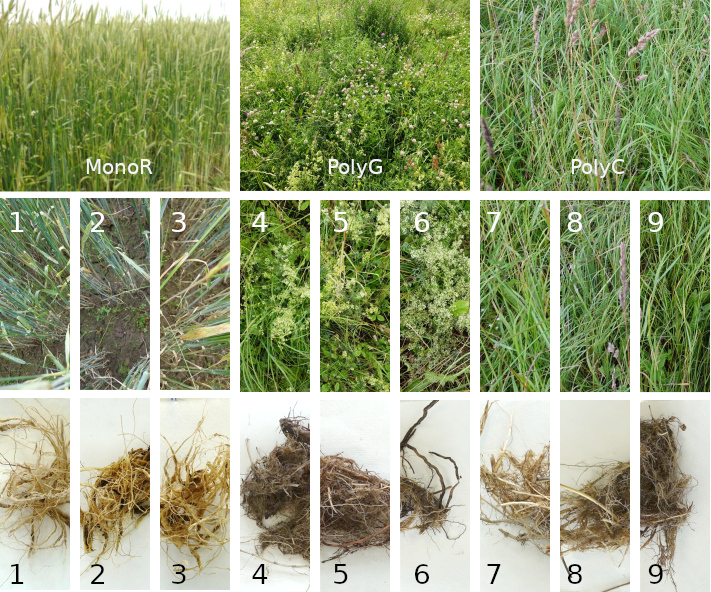

Supplement: Supplementary file 1 [file microorganisms-09-02339-s001.zip › S1_Figure_1.jpg]
